# Sleep, Emotion, and Sex-Specific Developmental Trajectories in Childhood and Adolescence

**DOI:** 10.3390/children13020171

**Published:** 2026-01-26

**Authors:** Giuseppe Marano, Marianna Mazza

**Affiliations:** 1 Department of Neuroscience, Head-Neck and Chest, Section of Psychiatry, Fondazione Policlinico Universitario Agostino Gemelli IRCCS, Largo Agostino Gemelli 8, 00168 Rome, Italy; 2 Department of Neuroscience, Section of Psychiatry, Università Cattolica del Sacro Cuore, 00168 Rome, Italy

**Keywords:** sleep, emotional regulation, sex differences, childhood, adolescence, neurodevelopment, puberty, anxiety and depression, externalizing behaviors, circadian rhythms

## Abstract

**Highlights:**

**What are the main findings?**

- Sleep–emotion interactions follow sex-specific developmental trajectories, with girls showing greater vulnerability to sleep-related internalizing symptoms and boys exhibiting more externalizing behaviors linked to sleep loss and circadian misalignment.
- Neurodevelopmental mechanisms (fronto-limbic maturation, REM sleep-dependent emotional processing, stress reactivity) interact with environmental factors to shape divergent emotional outcomes across childhood and adolescence.

**What is the implication of the main finding?**

- Sleep represents a critical, early, and modifiable target for identifying risk and preventing emotional dysregulation in pediatric populations.
- Sex-sensitive and developmentally informed interventions involving families, schools, and digital environments may enhance the effectiveness of pediatric mental health prevention and care.

**Abstract:**

Sleep plays a central role in shaping emotional development during childhood and adolescence, yet increasing evidence indicates that these processes unfold differently in boys and girls. This narrative review synthesizes current findings on sex-specific associations between sleep patterns, neurodevelopmental trajectories, and emotional regulation across pediatric populations. It examines how biological factors, including pubertal timing, sex hormones, circadian physiology, and maturation of fronto-limbic circuits, interact with environmental influences to generate distinct vulnerabilities to anxiety, depression, and behavioral dysregulation. Growing data suggest that girls exhibit greater sensitivity to sleep disturbances, particularly during the pubertal transition, with stronger links to internalizing symptoms such as anxiety and mood disorders. In contrast, boys appear more prone to externalizing behaviors and show differential responses to circadian misalignment and short sleep duration. Emerging evidence on sex-specific sleep architecture, REM-related emotional processing, and the bidirectional pathways through which sleep quality affects affective functioning are explored. Finally, clinical implications for early detection, personalized prevention, and targeted interventions tailored by sex and developmental stage are discussed. Understanding sex-based differences in sleep–emotion interactions offers a critical opportunity to refine pediatric mental health strategies and improve outcomes across developmental trajectories.

## 1. Introduction

Sleep is a fundamental biological process that plays a crucial role in physical growth, brain maturation, and emotional development during childhood and adolescence. Across pediatric development, sleep supports neural plasticity, emotional learning, and the regulation of affective responses, contributing to adaptive functioning and mental well-being [1,2,3]. Disruptions in sleep duration, quality, or timing during sensitive developmental periods have been consistently associated with emotional dysregulation, increased vulnerability to psychopathology, and impaired psychosocial functioning [4,5,6]. Childhood and adolescence are characterized by profound changes in sleep architecture and circadian regulation. Normative developmental trajectories include a gradual reduction in total sleep time, a shift toward later chronotypes, and marked modifications in rapid eye movement (REM) and slow-wave sleep, particularly during adolescence [7,8,9]. These sleep-related changes occur in parallel with the maturation of fronto-limbic neural circuits involved in emotion regulation, reward processing, and stress responsivity, suggesting a tightly interconnected relationship between sleep and emotional development [10,11].

Growing evidence indicates that these processes do not unfold uniformly across sexes. Sex differences in sleep patterns and emotional outcomes become increasingly pronounced from late childhood through adolescence, coinciding with pubertal maturation and hormonal changes [12,13]. Epidemiological studies show that, during adolescence, girls are more likely to experience sleep disturbances and internalizing symptoms such as anxiety and depression, whereas boys more frequently exhibit externalizing behaviors and impulse-control difficulties associated with short sleep duration and circadian misalignment [14,15,16]. These divergent trajectories may reflect sex-specific interactions between biological factors, including gonadal hormones and circadian physiology, and psychosocial stressors.

From a neurobiological perspective, sex differences in emotional vulnerability have been linked to differential maturation of prefrontal–amygdala circuitry, stress reactivity, and REM sleep-dependent emotional processing [17,18,19]. REM sleep, in particular, has been implicated in affective memory consolidation and emotional recalibration, processes that may be differentially sensitive to sleep disruption in boys and girls [20]. Alterations in REM sleep have been associated with heightened emotional reactivity and mood symptoms in pediatric populations, further underscoring the relevance of sleep architecture in emotional development [21].

Environmental and social factors also play a critical moderating role. School schedules, screen exposure, family routines, and sociocultural expectations interact with biological sex to shape sleep behaviors and emotional outcomes [22,23,24]. Importantly, the bidirectional nature of sleep–emotion relationships suggests that emotional distress may both result from and contribute to sleep disturbances, creating self-reinforcing cycles of vulnerability during development [25].

Despite increasing recognition of the importance of sleep for pediatric mental health, sex-specific mechanisms linking sleep and emotional regulation remain insufficiently integrated into developmental and clinical models. Understanding how sleep-related processes differentially influence emotional trajectories in boys and girls is essential for early identification of risk and the development of targeted, developmentally informed interventions. This narrative review aims to synthesize current evidence on sex-specific associations between sleep, neurodevelopment, and emotional regulation across childhood and adolescence, with a focus on clinical and preventive implications relevant to pediatric mental health.

## 2. Materials and Methods

### 2.1. Study Design

This article is a narrative review aimed at synthesizing current evidence on the relationships between sleep, emotional regulation, and sex-specific developmental trajectories in childhood and adolescence. A narrative approach was chosen to allow the integration of findings from heterogeneous study designs, including longitudinal cohort studies, neurodevelopmental research, and clinical investigations, which are not easily captured within a strictly systematic framework. Given the narrative design of the review, protocol registration (e.g., PROSPERO) and PRISMA reporting standards were not applicable. The narrative approach was intentionally adopted to allow conceptual integration of heterogeneous evidence across developmental, neurobiological, and clinical domains.

### 2.2. Literature Search Strategy

A comprehensive literature search was conducted in the electronic databases PubMed/MEDLINE, Scopus, and Web of Science to identify relevant peer-reviewed studies published up to June 2025. The search strategy combined keywords and Medical Subject Headings (MeSH) related to sleep, emotional development, and sex differences in pediatric populations. Search terms included combinations of: sleep, sleep disturbances, emotional regulation, emotion dysregulation, sex differences, gender differences, childhood, adolescence, puberty, anxiety, depression, and externalizing behaviors. Reference lists of selected articles and relevant reviews were manually screened to identify additional studies of interest. For narrative reviews, there is no fixed requirement regarding the number of databases searched. The selected databases were considered sufficient to capture high-impact literature in pediatric sleep, developmental neuroscience, and child psychiatry, and were complemented by manual screening of reference lists.

### 2.3. Eligibility Criteria

Studies were considered eligible if they met the following criteria: included children or adolescents (≤18 years); examined sleep characteristics (e.g., duration, quality, circadian timing, or sleep architecture); assessed emotional regulation, internalizing symptoms, or externalizing behaviors; reported sex-based or gender-based analyses, or provided data allowing sex-specific interpretation; and were published in English-language peer-reviewed journals. Experimental studies, observational studies, and longitudinal designs were included. Case reports and studies focusing exclusively on adult populations were excluded.

### 2.4. Data Extraction and Synthesis

Relevant information was extracted from each study, including sample characteristics, age range, sleep measures, emotional or behavioral outcomes, and reported sex differences. Given the heterogeneity of methodologies and outcome measures, a qualitative synthesis was performed rather than a meta-analytic approach. Findings were organized thematically, with particular attention to developmental stage (childhood versus adolescence) and sex-specific patterns of vulnerability. Given the substantial heterogeneity in study designs, populations, sleep measures, and emotional outcomes, a quantitative synthesis or meta-analysis was not methodologically appropriate for the aims of this review.

### 2.5. Methodological Considerations

In line with recommendations for narrative reviews, the present synthesis emphasizes conceptual integration and clinical relevance rather than exhaustive quantification of effect sizes. Potential sources of bias, including variability in sleep assessment methods and inconsistencies in sex-specific reporting, were considered when interpreting results. Reporting was informed by established guidance on narrative and integrative reviews in health sciences [26]. This approach is consistent with established guidance on narrative and integrative reviews, which emphasize conceptual synthesis and clinical relevance over exhaustive quantification when addressing complex developmental phenomena.

## 3. Sex Differences in Sleep Regulation Across Childhood and Adolescence

Sleep regulation undergoes profound developmental changes from childhood to adolescence, reflecting the maturation of circadian systems, homeostatic sleep pressure, and neuroendocrine processes. Increasing evidence indicates that these trajectories differ significantly between boys and girls, contributing to sex-specific patterns of sleep behavior and vulnerability to sleep disturbances [27,28,29].

### 3.1. Biological Foundations of Sex Differences in Sleep

Sex differences in sleep regulation emerge gradually during childhood and become more pronounced during adolescence, paralleling pubertal maturation. Puberty is associated with marked changes in gonadal hormone secretion, including rising levels of estrogen and progesterone in girls and testosterone in boys, which exert modulatory effects on circadian rhythms and sleep architecture [30,31]. Experimental and observational studies suggest that estrogen influences circadian phase timing and REM sleep expression, whereas progesterone has sedative and thermoregulatory effects that may alter sleep continuity [32]. Girls tend to report shorter sleep duration, poorer subjective sleep quality, and greater insomnia symptoms compared with boys during adolescence, even when objective sleep parameters are comparable [33]. These differences appear to be partly biologically driven, as girls generally experience earlier pubertal onset, which is associated with earlier circadian phase shifts and increased vulnerability to sleep disruption during school days [34].

### 3.2. Circadian Regulation and Chronotype Differences

Adolescence is characterized by a normative delay in circadian phase preference toward eveningness, a phenomenon influenced by both biological maturation and social factors [35]. Evidence suggests that girls may exhibit a stronger circadian sensitivity to pubertal and hormonal changes, whereas boys often show greater variability in sleep timing and a higher prevalence of extreme evening chronotypes [36].

Sex differences in circadian misalignment have important implications for emotional functioning. Eveningness and social jetlag have been more strongly associated with internalizing symptoms in adolescent girls, while in boys they are more frequently linked to risk-taking behaviors and externalizing problems [37,38]. These findings highlight the interaction between sex-specific circadian regulation and behavioral outcomes during development.

### 3.3. Sex-Specific Sleep Architecture

Beyond sleep duration and timing, sex differences have also been observed in sleep architecture. Polysomnographic studies indicate that girls generally exhibit higher slow-wave activity and more stable REM sleep across development, whereas boys show a steeper decline in slow-wave sleep during adolescence, reflecting differential cortical maturation processes [39,40]. REM sleep, which plays a key role in emotional processing and affective memory consolidation, may therefore represent a critical window through which sleep disturbances exert sex-specific effects on emotional regulation [19].

Alterations in REM sleep continuity and density have been associated with increased emotional reactivity and mood symptoms in pediatric populations, particularly among adolescent girls [41]. These findings support the hypothesis that sex-related differences in sleep architecture contribute to divergent emotional trajectories during development.

### 3.4. Interaction with Environmental and Psychosocial Factors

Biological sex interacts dynamically with environmental influences to shape sleep regulation. School start times, academic demands, screen exposure, and social stressors differentially affect boys and girls, often amplifying underlying biological vulnerabilities [27,42,43]. More broadly, pediatric sleep problems and clinically relevant sleep disorders are shaped by developmental factors but are also strongly influenced by behavioral and contextual determinants, supporting the need for developmentally informed assessment in children and adolescents [44]. Lifestyle disruptions and stress-related changes in daily routines, such as those described during large-scale societal stressors, may further affect sleep timing and quality, with downstream consequences for mental health and emotional well-being in youth [45]. Finally, the widespread exposure to social media has been increasingly linked to psychiatric and psychological outcomes in young people, and sleep disruption is frequently implicated as a plausible pathway connecting digital behaviors to affective and behavioral dysregulation during adolescence [46].

Sex-specific trajectories of sleep regulation across childhood and adolescence are associated with divergent patterns of emotional vulnerability. An overview of developmental stage-specific sleep characteristics and emotional outcomes in boys and girls is provided in Table 1.

## 4. Neurodevelopmental Pathways Linking Sleep and Emotion

Childhood and adolescence are characterized by rapid and non-linear brain maturation processes that are highly sensitive to sleep–wake regulation. Sleep plays a critical role in neurodevelopment by supporting synaptic pruning, myelination, and the functional integration of large-scale neural networks involved in emotional processing and regulation [47,48]. Disruptions in sleep during these sensitive developmental windows may therefore have lasting effects on emotional functioning and mental health vulnerability.

### 4.1. Maturation of Fronto-Limbic Circuits

Emotional regulation depends on the coordinated activity of fronto-limbic circuits, particularly connections between the prefrontal cortex and limbic structures such as the amygdala and hippocampus. During childhood and adolescence, these circuits undergo progressive structural and functional maturation, with the prefrontal cortex developing later than subcortical emotional systems [49,50]. This developmental imbalance contributes to heightened emotional reactivity and reduced regulatory control, especially during adolescence.

Sleep loss and sleep fragmentation have been shown to impair prefrontal cortical functioning and to exacerbate amygdala reactivity to emotional stimuli, leading to reduced top-down emotional regulation [51]. Neuroimaging studies in children and adolescents indicate that insufficient or irregular sleep is associated with altered functional connectivity within fronto–limbic networks, including reduced prefrontal regulation of amygdala reactivity and disrupted integration of emotion-processing circuits. These alterations have been linked to heightened emotional reactivity, impaired affective control, and increased vulnerability to internalizing and externalizing symptoms during development [27,52].

### 4.2. Role of REM Sleep in Emotional Processing

REM sleep is thought to play a central role in emotional memory consolidation and affective recalibration. Experimental evidence suggests that REM sleep facilitates the processing of emotionally salient experiences while attenuating their affective intensity, a mechanism that may be particularly important during development [53,54]. In children and adolescents, REM sleep occupies a larger proportion of total sleep time compared to adults, highlighting its potential relevance for emotional maturation. Alterations in REM sleep continuity, latency, or density have been associated with increased emotional reactivity, anxiety symptoms, and mood dysregulation in pediatric populations [55]. These associations appear especially pronounced during adolescence, a period marked by both changes in REM sleep organization and increased vulnerability to internalizing disorders. Sex-related differences in REM sleep expression may further modulate these effects, contributing to divergent emotional trajectories in boys and girls [22].

### 4.3. Sleep, Stress Reactivity, and Neuroplasticity

Sleep is closely intertwined with stress regulation systems, including the HPA axis. Adequate sleep supports adaptive stress responsivity, whereas chronic sleep disturbances are associated with heightened cortisol secretion and impaired stress recovery [56]. In developing individuals, repeated exposure to sleep-related stress may influence neuroplastic processes and bias emotional responses toward hyperarousal or negative affect.

Animal and human studies indicate that sleep deprivation during developmental periods can alter synaptic plasticity and stress-sensitive neural circuits, potentially increasing long-term vulnerability to anxiety and mood disorders [57]. These findings underscore the importance of sleep as a modulator of neurodevelopmental trajectories linking early-life experiences to later emotional outcomes [36].

### 4.4. Bidirectional Pathways Between Sleep and Emotional Development

Importantly, the relationship between sleep and emotional regulation is bidirectional. Emotional difficulties such as anxiety, depression, and behavioral dysregulation can disrupt sleep through mechanisms including hyperarousal, rumination, and dysregulated circadian rhythms [58,59]. Over time, this reciprocal interaction may give rise to self-perpetuating cycles in which sleep disturbances and emotional dysregulation reinforce one another, particularly during periods of developmental transition. Understanding the neurodevelopmental pathways linking sleep and emotion is therefore essential for identifying early markers of risk and informing preventive interventions aimed at promoting emotional resilience in children and adolescents.

**Figure 1:**
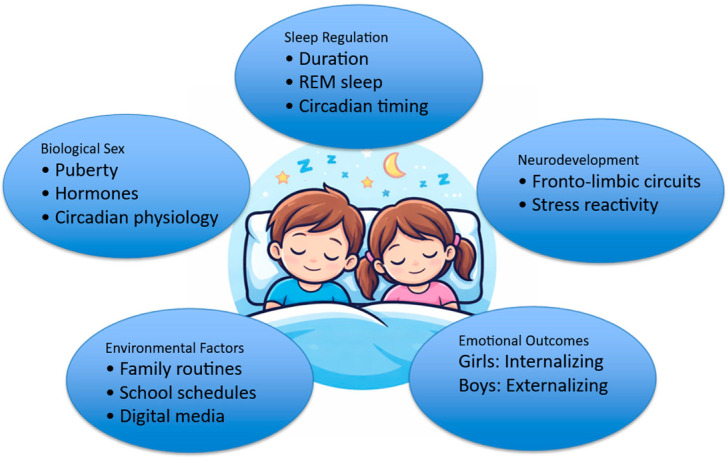
Sex-Specific Sleep–Emotion Pathways across Development. Note: Conceptual model illustrating sex-specific pathways linking sleep regulation, neurodevelopment, and emotional outcomes across childhood and adolescence. Biological maturation and environmental factors interact to shape sleep characteristics and emotional vulnerability, with divergent internalizing and externalizing trajectories in girls and boys.

An integrative conceptual model summarizing sex-specific sleep–emotion pathways across development is presented in Figure 1.

## 5. Sleep and Emotional Regulation: Divergent Trajectories in Boys and Girls

Accumulating evidence indicates that sleep disturbances are differentially associated with emotional and behavioral outcomes in boys and girls across childhood and adolescence. These divergent trajectories reflect the interaction between sex-specific neurodevelopmental processes, hormonal changes, and psychosocial influences, contributing to distinct patterns of vulnerability to internalizing and externalizing symptoms [60,61].

### 5.1. Internalizing Symptoms in Girls

From early adolescence onward, girls show a marked increase in internalizing symptoms, including anxiety, depressive mood, and emotional distress. Sleep disturbances, particularly insomnia symptoms, poor sleep quality, and circadian misalignment, have been consistently linked to these outcomes [62,63]. Longitudinal studies suggest that sleep problems often precede the onset of internalizing psychopathology in girls, supporting a potential causal role of sleep disruption in shaping emotional vulnerability [34].

Biological sensitivity to stress and heightened emotional reactivity may partly explain these associations. Girls tend to exhibit greater HPA axis reactivity and stronger emotional responses to interpersonal stressors, factors that may interact with sleep loss to amplify negative affect and rumination [64]. Moreover, pubertal timing appears to play a critical role, as early-maturing girls are particularly vulnerable to the combined effects of sleep disruption and emotional stress, increasing the risk for anxiety and mood disorders [65].

### 5.2. Externalizing Behaviors in Boys

In contrast, boys are more likely to exhibit externalizing behaviors such as impulsivity, hyperactivity, aggression, and risk-taking, which have been associated with insufficient sleep duration and irregular sleep–wake patterns [66,67]. Experimental and observational studies indicate that sleep restriction impairs executive functioning and inhibitory control, effects that may be more behaviorally expressed in boys through dysregulated or disruptive behaviors [53]. Circadian misalignment and eveningness have been linked to increased sensation seeking and conduct problems in male adolescents, suggesting that sleep timing, rather than sleep quality alone, may be particularly relevant for emotional and behavioral regulation in boys [68,69]. These findings underscore the importance of considering sex-specific behavioral manifestations of sleep-related emotional dysregulation.

### 5.3. Bidirectional and Developmental Considerations

Importantly, the associations between sleep and emotional regulation are bidirectional and evolve across development. Emotional difficulties may exacerbate sleep problems through mechanisms such as hyperarousal, worry, and dysregulated routines, while chronic sleep disturbances may impair emotional regulation capacities over time [5]. Sex differences in coping strategies and emotional expression further shape these reciprocal pathways, with girls more likely to internalize distress and boys more prone to externalize emotional difficulties. Recent developmental models emphasize dynamic and reciprocal interactions between sleep and emotional functioning, highlighting how sex differences in coping styles and stress reactivity may shape divergent trajectories over time [50].

These findings suggest that sleep disturbances contribute to sex-specific emotional trajectories during childhood and adolescence. Recognizing these divergent patterns is essential for early identification of at-risk individuals and for the development of tailored prevention and intervention strategies that account for both sex and developmental stage.

## 6. Environmental and Psychosocial Moderators of Sleep–Emotion Relationships

While biological maturation plays a central role in shaping sleep and emotional development, environmental and psychosocial factors critically moderate these processes across childhood and adolescence.

Family context, school-related demands, digital media exposure, and broader sociocultural influences interact with sex-specific biological vulnerabilities, amplifying or buffering the impact of sleep disturbances on emotional regulation [70,71]. Poor sleep quality is consistently associated with increased difficulties in emotion regulation in adolescents, not only through direct affective dysregulation but also via intermediary psychosocial processes such as daytime dysfunction and reduced self-control capacities, as recently demonstrated in a large school-based cohort study [72].

### 6.1. Family Environment and Daily Routines

Family routines and parenting practices strongly influence sleep behaviors in children and adolescents. Consistent bedtimes, parental monitoring, and emotionally supportive environments are associated with better sleep quality and more adaptive emotional functioning [73]. Conversely, family stress, irregular schedules, and inconsistent sleep-related practices have been linked to both sleep disturbances and emotional dysregulation, particularly in younger children [74]. Sex differences may emerge in how children respond to family-related stressors. Girls appear more sensitive to interpersonal and emotional aspects of the family environment, which may exacerbate sleep-related internalizing symptoms, whereas boys may be more affected by behavioral dysregulation associated with inconsistent routines [75].

### 6.2. School Schedules and Academic Demands

School-related factors represent a major external constraint on sleep, particularly during adolescence. Early school start times often conflict with adolescents’ biologically delayed circadian rhythms, leading to chronic sleep restriction and circadian misalignment [76]. These effects have been associated with poorer emotional regulation, increased depressive symptoms, and reduced stress tolerance.

Evidence suggests that girls may experience stronger associations between academic stress, sleep loss, and internalizing symptoms, whereas boys may show greater behavioral consequences, such as irritability and reduced impulse control, in response to insufficient sleep [42]. Longitudinal evidence suggests that the effects of sleep problems on externalizing behaviors are mediated by emotion dysregulation, highlighting an intermediary psychological mechanism that may be a target for early interventions [77].

School-based interventions aimed at promoting healthy sleep schedules have demonstrated benefits for both sleep duration and emotional well-being [78].

### 6.3. Screen Exposure and Digital Media Use

The widespread use of electronic devices and social media has emerged as a key environmental factor affecting sleep and mental health in youth. Screen exposure in the evening can delay sleep onset through both physiological mechanisms, such as blue-light-induced melatonin suppression, and psychological mechanisms, including cognitive and emotional arousal. Growing evidence links excessive or problematic social media use to sleep disturbances, anxiety, depressive symptoms, and emotional dysregulation in children and adolescents [79]. Current reviews indicate that disrupted sleep in adolescence is robustly associated with both depressive and anxiety symptomatology, underscoring its relevance in clinical assessment and intervention planning [80]. These associations may be particularly pronounced in girls, who tend to engage more in social and emotionally salient online interactions, whereas boys may be more affected by gaming-related sleep disruption. Sleep disruption has been proposed as a critical mediator linking digital behaviors to adverse emotional outcomes [81].

### 6.4. Sociocultural Stressors and Developmental Context

Broader sociocultural stressors, including peer relationships, societal expectations, and exposure to large-scale stressors, further shape sleep–emotion interactions. Changes in lifestyle and daily structure during periods of societal disruption have been shown to negatively affect sleep patterns and mental health in youth, highlighting the sensitivity of developing sleep–emotion systems to contextual instability [45].

It is clear that sleep-related emotional vulnerability cannot be understood in isolation from the environmental context in which children and adolescents develop. Integrating biological, psychological, and social perspectives is essential for identifying modifiable risk factors and informing prevention strategies aimed at promoting emotional well-being.

The interaction between neurodevelopmental mechanisms and environmental factors shapes sex-specific sleep–emotion pathways across development. An overview of key biological and contextual moderators, along with their clinical implications, is provided in Table 2.

## 7. Clinical and Preventive Implications

### 7.1. Early Identification and Risk Stratification

The evidence reviewed highlights sleep as a modifiable and developmentally sensitive target for promoting emotional well-being in children and adolescents. Sleep disturbances often emerge before the onset of clinically significant emotional or behavioral symptoms, making them a valuable early marker of vulnerability, as shown in longitudinal and clinical studies in pediatric populations [5,25]. Early sleep problems have been associated with later anxiety, depression, and behavioral dysregulation, supporting the role of sleep screening in preventive mental health strategies.

Screening approaches should be sensitive to sex-specific patterns, as girls are more likely to present with sleep-related internalizing symptoms, whereas boys more often exhibit behavioral dysregulation linked to insufficient or irregular sleep [29,33]. Integrating sleep assessment into pediatric and school-based mental health evaluations may therefore improve early risk stratification and diagnostic accuracy.

### 7.2. Sex-Sensitive and Developmentally Informed Interventions

The findings of this review underscore the importance of tailoring sleep-focused interventions to both developmental stage and sex-specific vulnerabilities. In girls, interventions targeting insomnia symptoms, rumination, and stress-related sleep disruption have been shown to reduce internalizing symptoms such as anxiety and depression [34]. In boys, strategies aimed at stabilizing sleep schedules and reducing circadian misalignment may be particularly effective in mitigating impulsivity and externalizing behaviors [42]. Behavioral sleep interventions, including sleep hygiene education and cognitive–behavioral approaches, are supported by a growing body of evidence in pediatric populations and may be enhanced by integrating emotion regulation strategies [2,3]. Such integrative approaches are consistent with developmental models of sleep–emotion interaction [82,83].

### 7.3. Role of Families, Schools, and Digital Environments

Family routines, parenting practices, and emotional climate play a central role in shaping sleep behaviors and emotional regulation in children [3,35]. Parent-focused interventions that promote consistent routines and appropriate limit-setting around screen use have been associated with improvements in both sleep quality and emotional functioning.

At the school level, evidence indicates that early school start times contribute to chronic sleep restriction and emotional distress in adolescents, supporting policy-level interventions such as delayed start times [76]. Educational initiatives aimed at increasing awareness of the importance of sleep for emotional well-being may further enhance prevention efforts [84].

Digital media exposure represents an increasingly relevant target for intervention. A growing literature links excessive social media use to sleep disturbances and adverse psychiatric outcomes in youth, with sleep disruption acting as a potential mediating mechanism [23,40,85]. Reducing evening screen exposure and promoting balanced digital habits may therefore represent key components of sleep-based preventive strategies.

Future research could also leverage signal-based and machine learning approaches, such as analyses of handwriting, graphomotor, and voice signals, to study complex behavioral patterns and emotional regulation in children and adolescents. These methodologies provide a promising framework to investigate sex-specific developmental trajectories and the impact of familial, school, and digital environments on sleep and emotional outcomes [86,87,88].

### 7.4. Implications for Public Health and Prevention

From a public health perspective, sleep represents a low-cost, scalable, and non-stigmatizing target for early intervention. Integrating sleep education and screening into existing pediatric mental health frameworks may help reduce the burden of emotional disorders and improve long-term outcomes [3,24]. A sex-sensitive and developmentally informed approach to sleep and emotional regulation offers an opportunity to refine preventive strategies and to support more personalized models of pediatric mental health care.

## 8. Conclusions

Sleep plays a central and developmentally sensitive role in shaping emotional regulation and mental health trajectories during childhood and adolescence. The evidence synthesized in this review highlights that sleep–emotion interactions are not uniform across development but follow sex-specific pathways influenced by biological maturation, neurodevelopmental processes, and environmental context.

Girls appear to show greater vulnerability to sleep-related internalizing symptoms, particularly during the pubertal transition, whereas boys more frequently exhibit externalizing behaviors associated with insufficient sleep and circadian misalignment. These divergent trajectories reflect the interplay between sex hormones, maturation of fronto-limbic circuits, stress reactivity, and psychosocial influences, underscoring the importance of adopting a sex-sensitive perspective in pediatric sleep and mental health research.

Importantly, the bidirectional nature of sleep and emotional regulation suggests that sleep disturbances may both precede and exacerbate emotional difficulties, creating self-reinforcing cycles of vulnerability across development [89]. This highlights sleep as a critical and modifiable target for early identification, prevention, and intervention in pediatric mental health [90,91].

From a clinical and public health perspective, integrating sleep assessment and education into pediatric care, school-based programs, and preventive mental health strategies represents a promising approach to improving emotional well-being in youth. Interventions that are developmentally informed and tailored to sex-specific patterns of vulnerability may enhance effectiveness and contribute to more personalized models of care.

Ongoing research should prioritize longitudinal and mechanistic studies that clarify causal pathways linking sleep, neurodevelopment, and emotional outcomes, while accounting for sex differences and environmental moderators. Advancing this line of inquiry has the potential to refine early prevention strategies and to support healthier emotional development across childhood and adolescence.

## Figures and Tables

**Table 1:** Sex-Specific Sleep Characteristics and Emotional Outcomes across Development.

Developmental Stage	Sleep Characteristics	Girls	Boys	Emotional/Behavioral Outcomes
Childhood	Relatively stable sleep architecture; gradual reduction in total sleep time	Slightly better objective sleep continuity; higher emotional sensitivity	Greater variability in sleep timing	Subtle sex differences; early emotional sensitivity may emerge in girls
Early Adolescence	Puberty-related circadian delay; changes in REM and slow-wave sleep	Earlier pubertal timing; increased insomnia symptoms; higher REM stability	Greater eveningness and irregular sleep–wake patterns	Girls: rising anxiety and mood symptoms; Boys: impulsivity, hyperactivity
Mid–Late Adolescence	Chronic sleep restriction; circadian misalignment with school schedules	Poorer subjective sleep quality; higher internalizing vulnerability	Short sleep duration; pronounced social jetlag	Girls: depression, emotional distress; Boys: externalizing behaviors, risk-taking
Key Moderators	Hormonal changes, stress reactivity, REM sleep processing	Higher HPA axis reactivity; stronger emotional arousal	Greater behavioral expression of sleep loss	Divergent emotional trajectories shaped by sex and context

Abbreviations: REM, rapid eye movement; HPA, hypothalamic–pituitary–adrenal.

**Table 2:** Neurobiological and Environmental Pathways Linking Sleep and Emotional Regulation: Sex-Specific Considerations.

Domain	Mechanism	Girls	Boys	Clinical Implications
Neurodevelopment	Fronto-limbic circuit maturation	Prolonged emotional sensitivity due to later prefrontal regulatory control	Greater behavioral disinhibition during sleep loss	Identification of sex-specific windows of vulnerability
REM Sleep	Emotional memory consolidation and affective recalibration	REM alterations linked to anxiety and mood dysregulation	REM disruption linked to behavioral emotional reactivity	REM sleep as a target for early detection and prevention
Stress Regulation	HPA axis reactivity	Higher cortisol reactivity to interpersonal stress	Greater behavioral expression of stress-related sleep loss	Stress-informed and sex-sensitive interventions
Family Context	Daily routines and emotional climate	Higher sensitivity to relational stress	Greater impact of inconsistent structure	Parent-focused preventive strategies
School & Circadian Factors	Early start times and circadian misalignment	Stronger links to internalizing symptoms	Stronger links to irritability and impulse control	Support for delayed school start policies
Digital Media	Evening screen exposure and cognitive arousal	Social comparison and emotional arousal	Gaming-related sleep disruption	Sleep-focused digital hygiene interventions

Abbreviations: REM, rapid eye movement; HPA, hypothalamic–pituitary–adrenal.

## Data Availability

No new data were created.
